# The relationship between autoimmune thyroid disease, thyroid nodules and sleep traits: a Mendelian randomization study

**DOI:** 10.3389/fendo.2023.1325538

**Published:** 2024-03-18

**Authors:** Suijian Wang, Kui Wang, Xiaohong Chen, Shaoda Lin

**Affiliations:** ^1^ Department of Endocrinology, The First Affiliated Hospital, School of Medicine, Shantou University, Shantou, China; ^2^ Department of Gastroenterology, Ruijin Hospital, School of Medicine, Shanghai Jiao Tong University, Shanghai, China

**Keywords:** thyroid nodules, Graves’ disease, hypothyroidism, Mendelian randomization, GWAS

## Abstract

**Background:**

Previous studies have suggested a potential association between Autoimmune thyroid disease Thyroid nodules and Sleep Traits, but the evidence is limited and controversial, and the exact causal relationship remains uncertain.

**Objective:**

Therefore, we employed a MR analysis to investigate the causal relationship between Autoimmune thyroid disease, Thyroid nodules and Sleep Traits.

**Methods:**

To explore the interplay between Autoimmune thyroid disease Thyroid nodules and Sleep Traits, we employed MR studies utilizing summary statistics derived from GWAS in individuals of European ancestry. To ensure robustness, multiple techniques were employed to assess the stability of the causal effect, including random-effect inverse variance weighted, weighted median, MR-Egger regression, and MR-PRESSO. Heterogeneity was evaluated using Cochran’s Q value. Additionally, we investigated the presence of horizontal pleiotropy through MR-Egger regression and MR-PRESSO.

**Results:**

The IVW method indicates a significant causal relationship between “Getting up” and autoimmune hypothyroidism, as revealed by the IVW method (OR: 0.59, 95% CI: 0.45 to 0.78, P-value = 1.99e-4). Additionally, there might be a potential correlation between sleep duration and autoimmune hypothyroidism (OR: 0.76, 95% CI: 0.60 to 0.79, P-value = 0.024). Moreover, the observed potential positive link between daytime nap and thyroid nodules (OR: 1.66, 95% CI: 1.07 to 2.58, P-value = 0.023) is subject to caution, as subsequent MR PRESSO testing reveals the presence of horizontal pleiotropy, raising concerns about the reliability of the findings. The findings suggested a potential inverse association between Autoimmune hypothyroidism and Getting up (OR: 0.99, 95% CI: 0.98 to 1.00, P-value = 6.66e-3).As the results of MR-Egger method(OR: 1.00, 95% CI: 0.98 to 1.02, P-value = 0.742) exhibited an opposing trend to that observed with the IVW method and the results did not reach significance after P-value correction.

**Conclusion:**

The results of our study reveal a notable cause-and-effect relationship between Getting up and Autoimmune hypothyroidism, indicating its potential role as a protective factor against this condition. However, no causal connection was observed between sleep traits and Graves’ disease or Thyroid nodules

## 1 Introduction

Sleep plays a crucial role in maintaining human health, impacting various vital physiological functions (1, 2). The regulation of sleep is intricate and subject to precise physiological control (3). Virtually all cells of the body possess an intrinsic circadian clock that spans approximately 24 hours, operating autonomously (4). This circadian system plays a crucial role in tightly regulating physiological functions and endocrine rhythms (4, 5). A wealth of evidence concurs that chronic circadian disruption, arising from either shift work or crossing time zones frequently, yields lasting repercussions on human health, heightening the risk of mental disorders, type 2 diabetes mellitus, cardiovascular disease, and mortality (6–10). The disruption of the endocrine system’s circadian regulation represents a crucial mechanism through which these adverse outcomes associated with circadian disturbances are mediated (11–13).

Autoimmune thyroid disease(AITD) serves as the leading cause of acquired thyroid dysfunction, exhibiting two distinct clinical presentations known as hyperthyroidism (Graves’ disease) and hypothyroidism (Hashimoto’s thyroiditis) (14). Hashimoto’s thyroiditis is characterized by the presence of autoantibodies targeting thyroid peroxidase and thyroglobulin (15). In contrast, Graves’ disease is associated with the detection of autoantibodies against the TSH receptor (15). Twin studies have provided an estimation indicating that genetic factors contribute to 70%–80% of the susceptibility to autoimmune thyroid disease (16, 17). Multiple susceptibility genes, along with epigenetic and environmental influences, collectively contribute to the development of both conditions (18, 19).

Previous observational studies have proposed that a shorter sleep duration may serve as an independent risk factor for subclinical hypothyroidism and thyroid nodules (14, 20). Conversely, other research suggests that subclinical hypothyroidism may be the underlying cause of reduced sleep duration (21). However, it is important to acknowledge that these findings are subject to the potential confounding effect inherent in observational studies, which may introduce reverse causation. Limited research has been conducted on the relationship between sleep characteristics and hyperthyroidism, but it is widely accepted within the current literature that the heightened neural excitability resulting from thyrotoxicosis, a consequence of hyperthyroidism, is the primary cause of insomnia in individuals with this condition (15). Nonetheless, further investigation is necessary to determine whether hyperthyroidism itself is genuinely associated with sleep disturbances. To date, no studies have provided additional evidence in this particular aspect, and providing a definitive answer to this question through clinical research poses significant difficulties.

The association between sleep characteristics and AITD, as either an inherent correlation or a potential risk factor, is still a subject of debate. In order to address this issue, we undertook a bidirectional Mendelian randomization (MR) study, utilizing genetic variants identified from genome-wide association studies (GWAS) as instrumental variables (IVs) for environmental exposure (22). This unique methodology allows for causal inference between exposure and outcome, enabling us to elucidate the causal link between AITD and sleep traits.

## 2 Materials and methods

### 2.1 Study design and the assumption of MR

**Figure 1:**
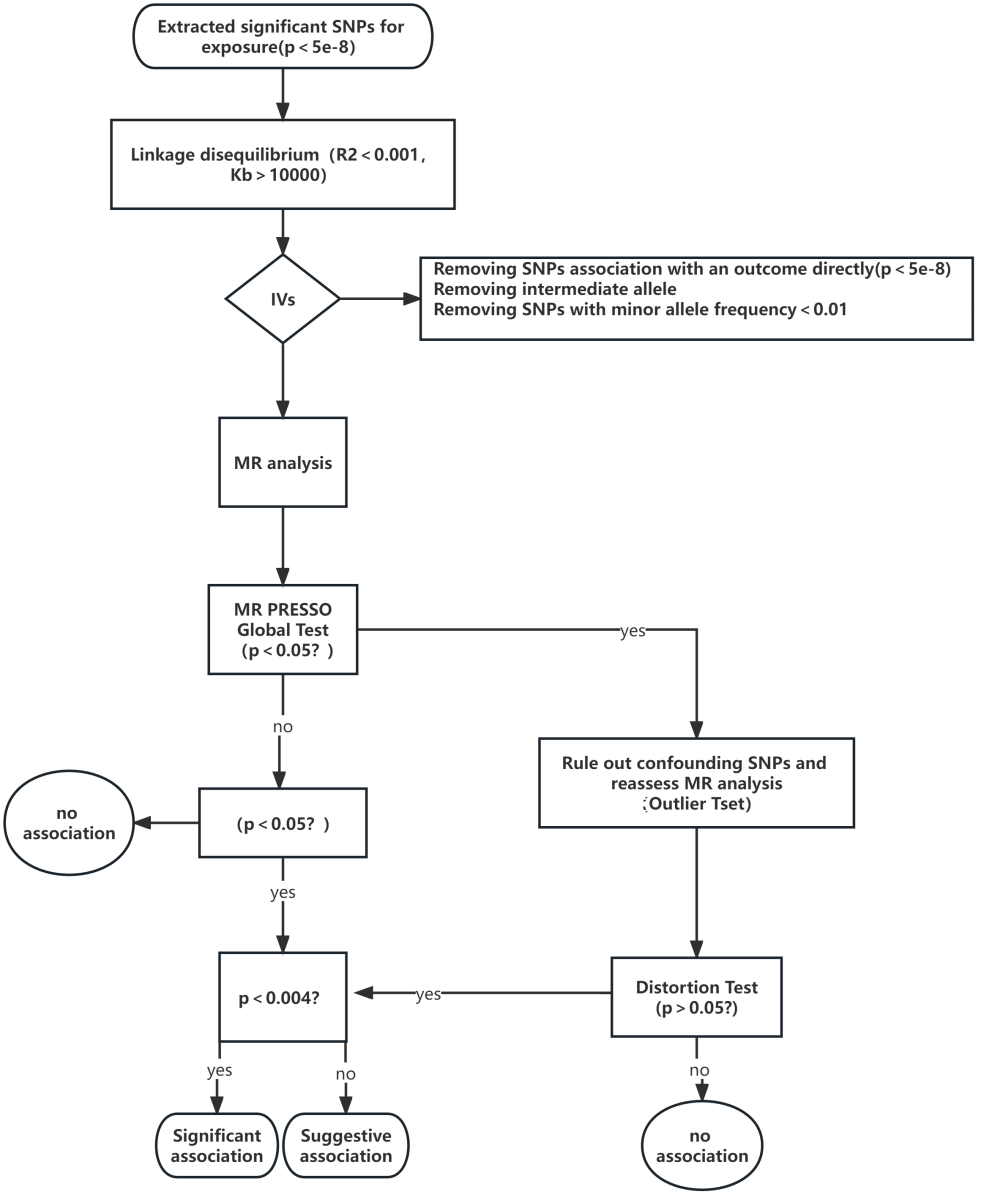
A flow chart outlining the study design and the steps involved in MR analysis.

In order to investigate the potential association between Graves disease, Autoimmune hypothyroidism, and six sleep traits (Daytime nap, Getting up, Sleep duration, Daytime sleepiness, Short sleep, Long sleep, and Insomnia), we employed a two-sample MR design. Our primary objective was to assess the overall effects and determine the link between these conditions and sleep characteristics. To gain further insights into the bidirectional impact of Graves disease and autoimmune hypothyroidism on sleep traits, we performed reverse MR analysis, as depicted in **Figure 1**. It is important to note that our MR analysis relied on several key assumptions: (i) the single nucleotide polymorphisms (SNPs) selected as IVs were derived from GWAS and exhibited significant associations with the aforementioned exposures; (ii) the IVs were not influenced by any potential confounding factors; (iii) the IVs manifested a direct influence solely on the risk of outcomes through the investigated exposures (23).

### 2.2 Data sources

Daytime nap Hassan S Dashti et al. conducted a genome-wide association analysis on self-reported daytime napping using data from the UK Biobank, which consisted of 452,633 individuals. The assessment of daytime napping in the UK Biobank involved the utilization of a questionnaire, whereby participants were asked to provide information regarding their napping habits. This variable was treated as a frequency measure and categorized into three response options: never/rarely, usually, and always. Within the cohort of UK Biobank participants with European ancestry (N = 452,633), it was found that 38.2% and 5.3% of individuals reported occasional and consistent napping, respectively (24).

Getting up in the Morning Samuel E. Jones et al. employed a genome-wide association analysis to investigate the relationship between self-reported chronotype and genetic variations (25). The analysis included 11,977,111 imputed variants in 449,734 individuals of European ancestry from the UK Biobank. The determination of an individual’s innate preference for morning or night activities was ascertained within the study. Out of the total surveyed population, which included 135,447 participants who completed at least one survey, it was observed that 75.5% of individuals were categorized as either morning or night persons based on their responses. Participants who provided neutral responses (N=32,842) or conflicting answers (N=309) were excluded from the analysis (26). To enhance the statistical power, they also performed a meta-analysis, merging the summary statistics from a separate GWAS focused on self-reported morningness.This secondary analysis encompassed 11,947,421 variants in 248,098 individuals of European ancestry from the 23andMe research cohort (26, 27).

Sleep duration Hassan S Dashti et al. performed analyses of self-reported measures of sleep (28). Self-reported measures analyzed included (a) the number of hours spent sleeping over a 24-h period (including naps); (b) insomnia; (c) chronotype—where “definitely a ‘morning’’ person”, “more a ‘morning’’ than ‘evening’’ person”, “more an ‘evening’’ than a ‘morning’’ person”, “definitely an ‘evening’’ person” and “do not know”, were coded as 2, 1, −1, -2 and 0, respectively, in our continuous variable (29).

Within the cohort of UK Biobank participants with European ancestry (N = 446,118), the average self-reported habitual sleep duration was 7.2 hours per day, with a standard deviation of 1.1. Subsequent analyses were conducted separately for individuals with short sleep duration (<7 hours; N = 106,192 cases) and long sleep duration (≥9 hours; n = 34,184 cases), relative to those with a reference sleep duration of 7-8 hours (N = 305,742 controls). These analyses revealed the identification of 27 loci associated with short sleep duration and 8 loci associated with long sleep duration. Importantly, 13 of these loci were found to be independent from the previously reported 78 loci associated with sleep duration (28).

Daytime sleepiness

A total of 452,071 individuals with European genetic ancestry from the UK Biobank dataset participated in this study (30). These individuals were asked to provide self-reported data on the frequency of daytime sleepiness, specifically relating to instances where they unintentionally dozed off or fell asleep during the day (e.g., while working, reading, or driving). Response categories included “never” (N = 347,285), “sometimes” (N = 92,794), “often” (N = 11,963), and “all of the time” (N = 29). Furthermore, the analysis revealed that the severity of daytime sleepiness exhibited a positive association with older age, female sex, higher body mass index (BMI), various behavioral, social, and environmental factors, as well as the presence of chronic diseases (29).

Insomnia Jacqueline M. Lane et al. conducted a comprehensive investigation wherein they successfully identified 57 loci associated with self-reported insomnia symptoms within the UK Biobank population (N = 453,379),participants were asked to self-report their experience of insomnia symptoms by responding to the question, “Do you have trouble falling asleep at night or do you wake up in the middle of the night?”. Analysis of this sample revealed that 29% of individuals reported experiencing frequent insomnia symptoms (“usually”). Interestingly, a higher prevalence of these symptoms was observed among women (32% vs. 24%), as well as in older participants, shift workers, and those with shorter self-reported sleep duration (31).

The FinnGen Biobank, consisting of individuals with European ancestry, has provided valuable data for a GWAS pertaining to ATID (32). The GWAS dataset included 4,462 cases and 320,703 controls for Graves’ disease (diagnosed with ICD-10 code E05.0), comprising 3,726 female patients and 736 male patients. Additionally, the dataset for autoimmune hypothyroidism included 40,926 cases and 274,069 controls (diagnosed with ICD-10 code E06.3), consisting of 32,732 female patients and 8,194 male patients. With regards to thyroid nodules, a total of 9485 cases and 367792 controls were included in the dataset, classified under the diagnostic code ICD-10 E04.1. Out of these, there were 7899 female patients and 1586 male patients. The complete information is in **Table 1**.

**Table 1:** Forward MR of the correlation between sleep and hypothyroidism.

(exposure)	(outcome)	Nsnp	Methods	Beta	SE	Beta/OR (95%CI)	*P* value	Heterogeneity*Q_P* value	Horizontal pleiotrop	
									Egger intercept P	*MRPresso P*
Daytime nap	Hypothyroidism	88	MR-Egger	-1.12	0.49	0.32 (0.12-0.85)	0.024	2.40E-06	0.026	0.110
			Weighted median	0.05	0.16	1.06 (0.77-1.47)	0.728			
			Inverse variance weighted	-0.05	0.13	0.94 (0.72-1.24)	0.676			
Getting up		72	MR-Egger	-0.08	0.54	0.92 (0.32-2.67)	0.876	1.62E-10	0.409	0.633
			Weighted median	-0.57	0.14	0.56 (0.43-0.74)	4.53e-5			
			Inverse variance weighted	-0.52	0.14	0.59 (0.45-0.78)	1.99e-4			
Sleep duration		73	MR-Egger	-0.11	0.47	0.89 (0.35-2.27)	0.808	3.02E-19	0.740	0.308
			Weighted median	-0.27	0.11	0.76 (0.61-0.94)	0.011			
			Inverse variance weighted	-0.27	0.12	0.76 (0.60-0.97)	0.024			
Daytime sleepiness		38	MR-Egger	-0.24	1.71	0.79(0.03-22.82)	0.889	1.45E-13	0.899	0.897
			Weighted median	0.13	0.30	1.14(0.63- 2.07)	0.664			
			Inverse variance weighted	-0.02	0.38	0.97(0.46- 2.06)	0.941			
Short sleep		25	MR-Egger	-0.85	1.78	0.42(0.01-14.03)	0.635	0.197	0.509	0.186
			Weighted median	0.35	0.39	1.43 (0.66- 3.10)	0.370			
			Inverse variance weighted	0.32	0.31	1.38 (0.74- 2.55)	0.307			
long sleep		7	MR-Egger	1.16	4.37	3.21 (0.00-1702.17)	0.800	0.004	0.630	0.397
			Weighted median	-1.24	1.10	0.29 (0.03- 2.52)	0.260			
			Inverse variance weighted	-0.94	1.36	0.39(0.03- 5.67)	0.489			
Insominia		40	MR-Egger	0.14	0.54	1.16 (0.40-3.34)	0.790	0.008	0.983	0.438
			Weighted median	-0.02	0.22	0.97 (0.63-1.50)	0.893			
			Inverse variance weighted	0.15	0.17	1.17 (0.83-1.64)	0.370			

### 2.3 Selection of genetic instrumental variables

To satisfy the assumption of a strong correlation between the exposure and single nucleotide polymorphisms (SNPs), we adopted a rigorous genome-wide significance threshold of P-value (P-value<5x10^-8^) when selecting instrumental variables (IVs). Moreover, to achieve dataset harmonization, we systematically removed variants displaying potential linkage disequilibrium (r^2 ^= 0.001, 10,000 kb). Subsequent steps involved standardizing the effect estimates for both the exposure and outcome variants, along with the removal of any potential SNPs harboring incompatible alleles or palindromic SNP (33).We utilized the PhenoScanner database (http://www.phenoscanner.medschl.cam.ac.uk/phenoscanner) to investigate the selected instrumental variables associated with other phenotypes that could potentially influence the outcome. when considering Graves’ disease and Autoimmune hypothyroidism as the exposures, we excluded several SNPs (rs568999, rs2160232, rs9271500, rs10995256) that were associated with anxiety and inflammatory bowel disease, as previous studies have suggested a link between anxiety, inflammatory bowel disease, and sleep disorders (34–37). To assess the strength of genetically determined IVs and avoid any bias towards weak IVs, we used F statistics (beta2/se2) (38) and ensured that F>10 in line with the first MR assumption (39, 40).

### 2.4 Mendelian randomization analyses

The primary analysis employed the inverse-variance weighted (IVW) method using a random-effects model, taking into consideration the variation among SNPs (41). To validate the findings of the primary analysis, several sensitivity analyses were conducted. Moreover, we employed the weighted median (WM) method, which necessitates allocating over 50% of the weight to valid IVs, to estimate the causal effects (42). Additionally, we evaluated possible horizontal pleiotropy using MR-Egger intercepts (42, 43). To capture and resolve any potential horizontal pleiotropic outliers, we implemented the MR-PRESSO framework and adjusted the IVW estimate by eliminating outliers (44). Furthermore, we conducted a leave-one-out analysis to investigate whether the effect estimates were impacted by any singular outlier variant. The analyses were conducted using the R software (version 4.2.3) Two-Sample MR package. To account for multiple testing, we implemented a Bonferroni-corrected threshold of P-value < 0.0023 (P < 0.05/7/3). P-values ranging from 0.0023 to 0.05 were considered indicative of suggestive associations. The MR analysis procedure is visually presented in **Figure 1** through a flowchart, outlining each step in a sequential manner.

## 3 Result

### 3.1 Forward MR analysis

#### 3.1.1 Sleep traits and autoimmune hypothyroidism

After the process of data screening, we extracted a total of 88 SNPs as IVs from Daytime nap, 72 SNPs from Getting up, 73 SNPs from Sleep duration, 38 SNPs from Daytime sleepiness, 25 SNPs from Short sleep, 7 SNPs from long sleep, and 40 SNPs from Insomnia when considering Autoimmune hypothyroidism as the outcome (**Supplementary Tables 1**, **2**). Upon evaluating the influence of 72 SNPs from Getting up on Autoimmune hypothyroidism, a consistent assessment revealed a significant negative correlation between Getting up and Autoimmune hypothyroidism (OR: 0.59, 95% CI: 0.45 to 0.78, P-value = 1.99e-4, **Table 2**). This consistent direction of effect persisted when utilizing both the MR-Egger (OR: 0.92, 95% CI: 0.32 to 2.67, P-value = 0.876, **Table 2**) and Weighted Median methods (OR: 0.56, 95% CI: 0.43 to 0.74, P-value = 4.53e-5, **Table 2**). Subsequent tests indicated the presence of heterogeneity (Q-P-value = 1.62E-10, **Table 2**), prompting the adoption of a random-effects model for estimating the magnitude of the MR effect. The MR Egger intercept (Egger intercept P-value = 0.409,**Table 2**) confirmed the absence of horizontal pleiotropy. Upon conducting further MR PRESSO testing, three outlier SNPs were identified, and their exclusion resulted in consistent findings with the original results (Distortion Test P-value = 0.633, **Table 2**). Similarly, when investigating the impact of 73 SNPs on sleep duration in relation to Autoimmune hypothyroidism, a negative correlation was found (OR: 0.76, 95% CI: 0.60 to 0.79, P-value = 0.024, **Table 2**). This consistent relationship persisted when utilizing both the MR-Egger(OR: 0.89, 95% CI: 0.35 to 2.27, P-value = 0.808, **Table 2**) and Weighted Median methodologies(OR: 0.76, 95% CI: 0.61 to 0.94, P-value = 0.011, **Table 2**). Subsequent analyses indicated the presence of heterogeneity (Q-P-value = 3.02E-19,**Table 2**), prompting the utilization of a random-effects model to estimate the magnitude of the MR effect. The MR Egger intercept (Egger intercept P-value = 0.740, **Table 2**) confirmed the absence of horizontal pleiotropy. Further application of MR PRESSO analysis revealed the presence of six atypical SNPs. However, after their removal, the obtained results remained unchanged compared to the initial findings(Distortion Test P-value = 0.308, **Table 2**). However, after Bonferroni correction, the results were not statistically significant. Additionally, no significant associations were observed between Daytime nap (OR: 0.94, 95% CI: 0.72 to 1.24,P-value = 0.676, **Supplementary Table 2, Table 1**), Daytime sleepiness (OR: 0.97, 95% CI: 0.46 to 2.06, P-value = 0.941, **Supplementary Table 2, Table 1**), Short sleep (OR: 1.38, 95% CI: 0.74 to 2.52, P-value = 0.307, **Supplementary Table 2, Table 1**), Long sleep (OR: 0.39, 95% CI: 0.03 to 5.67, P-value = 0.489, **Supplementary Table 2, Table 1**), and Insomnia (OR: 1.17, 95% CI: 0.83 to 1.64, P-value = 0.370, **Supplementary Table 2, Table 1**) and Autoimmune hypothyroidism.

**Table 2:** Reverse MR of the correlation between sleep and hypothyroidism.

(exposure)	(outcome)	Nsnp	Methods	Beta	SE	Beta/OR (95%CI)	*P* value	Heterogeneity*Q_P* value	Horizontal pleiotrop	
									Egger intercept P	*MRPresso P*
Hypothyroidism	Daytime nap	152	MR-Egger	7.27e-3	7.69e-3	1.01 (0.99-1.02)	0.346	8.47e-7	0.653	0.838
			Weighted median	7.20e-3	3.20e-3	1.01 (1.00-1.01)	0.024			
			Inverse variance weighted	4e-3	2.54e-3	1.00 (1.00-1.01)	0.115			
	Getting up	152	MR-Egger	1.53e-3	0.01	1.00 (0.98-1.02)	0.742	3.90e-10	0.194	0.503
			Weighted median	-2.85e-3	4.48e-3	1.00 (0.99-1.01)	0.524			
			Inverse variance weighted	-9.68e-3	3.57e-3	0.99 (0.98-1.00)	6.66e-3			
	Sleep duration	148	MR-Egger	7.05e-3	0.01	1.01 (0.98-1.04)	0.647	2.86e-10	0.652	0.703
			Weighted median	9.6e-4	6.28e-3	1.00 (0.99-1.01)	0.878			
			Inverse variance weighted	5.07e-4	5.16e-3	1.00 (0.99-1.01)	0.921			
	Daytime sleepiness	148	MR-Egger	9.05e-3	6.5e-3	1.01 (1.00-1.02)	0.165	1.59e-6	0.370	0.674
			Weighted median	4.73e-3	3.04e-3	1.00 (1.00-1.01)	0.120			
			Inverse variance weighted	3.55e-3	2.18e-3	1.00 (1.00-1.01)	0.103			
	Short sleep	148	MR-Egger	-2.5e-4	6.25e-3	1.00 (0.99-1.01)	0.968	7.29e-8	0.748	0.394
			Weighted median	2.37e-3	2.76e-3	1.00 (1.00-1.01)	0.391			
			Inverse variance weighted	1.64e-3	2.09e-3	1.00 (1.00-1.01)	0.432			
	long sleep	148	MR-Egger	2.97e-3	3.96e-3	1.00 (1.00-1.01)	0.453	0.035	0.825	0.046
			Weighted median	2.24e-3	1.95e-3	1.00 (1.00-1.01)	0.250			
			Inverse variance weighted	2.15e-3	1.32e-3	1.00 (1.00-1.00)	0.104			
	Insominia	148	MR-Egger	-7.56e-3	8.77e-3	0.99 (0.98-1.01)	0.389	8.47e-7	0.242	0.165
			Weighted median	5.1e-4	4.06e-3	1.00 (0.99-1.01)	0.900			
			Inverse variance weighted	2.13e-3	2.94e-3	1.00 (1.00-1.01)	0.468			

#### 3.1.2 Sleep traits and Graves’ disease

Following data screening procedures, a comprehensive selection of IVs was obtained. Specifically, we identified 87 SNPs from Daytime nap, 72 SNPs from Getting up, 73 SNPs from Sleep duration, 38 SNPs from Daytime sleepiness, 25 SNPs from Short sleep, 7 SNPs from long sleep, and 40 SNPs from Insomnia for the evaluation of Graves’ disease as the primary outcome(**Supplementary Tables 1**, **2**). In the positive MR analysis, no significant causal relationship was found between Daytime nap (OR: 1.32, 95% CI: 0.65 to 2.67, P-value = 0.441, **Supplementary Table 2, Table 3**), Getting up (OR: 0.69, 95% CI: 0.39 to 1.23, P-value = 0.205, **Supplementary Table 2**), Sleep duration (OR: 0.79, 95% CI: 0.50 to 1.26, P-value = 0.323, **Supplementary Table 2**, **Table 3**), Daytime sleepiness (OR: 0.83, 95% CI: 0.15 to 4.74, P = 0.834, **Supplementary Table 2**, **Table 3**), Short sleep (OR: 2.61, 95% CI: 0.04 to 159.44, P-value = 0.647, **Supplementary Table 2**, **Table 3**), Long sleep (OR: 0.08, 95% CI: 0.00 to 2.71e6, P-value = 0.932, **Supplementary Table 2**, **Table 3**), and Insomnia (OR: 1.96, 95% CI: 0.94 to 4.08, P-value = 0.073, **Supplementary Table 2**, **Table 3**) with Graves’ disease.

**Table 3:** Forward MR of the correlation between sleep and graves.

(exposure)	(outcome)	Nsnp	Methods	Beta	SE	Beta/OR (95%CI)	*P* value	Heterogeneity*Q_P* value	Horizontal pleiotrop	
									Egger intercept P	*MRPresso P*
Daytime nap	graves	87	MR-Egger	0.98	1.30	2.68(0.21-34.43)	0.450	1.96e-4	0.572	0.766
			Weighted median	0.42	0.41	1.53 (0.68- 3.43)	0.307			
			Inverse variance weighted	0.27	0.35	1.32 (0.65- 2.67)	0.441			
Getting up		72	MR-Egger	1.61	1.11	5.03(0.57-44.69)	0.151	0.013	0.069	0.690
			Weighted median	-0.53	0.37	0.58 (0.28- 1.21)	0.148			
			Inverse variance weighted	-0.37	0.29	0.69 (0.39- 1.23)	0.205			
Sleep duration		73	MR-Egger	-0.60	0.93	0.55 (0.09-3.46)	0.524	2.45e-4	0.687	0.787
			Weighted median	-0.58	0.30	0.56 (0.31-1.01)	0.052			
			Inverse variance weighted	-0.23	0.23	0.79 (0.50-1.26)	0.323			
Daytime sleepiness		38	MR-Egger	-0.11	3.99	0.89(0.00-2232.77)	0.977	1.43e-8	0.985	0.121
			Weighted median	0.27	0.77	1.32(0.29- 6.08)	0.720			
			Inverse variance weighted	-0.18	0.88	0.83(0.15- 4.74)	0.834			
Short sleep		25	MR-Egger	-5.61	11.93	0.00 (0.00-5.29e7)	0.647	3.07e-25	0.581	0.185
			Weighted median	-0.72	1.18	0.48 (0.05- 4.97)	0.541			
			Inverse variance weighted	0.95	2.09	2.61 (0.04- 159.44)	0.647			
long sleep		7	MR-Egger	-2.53	8.85	1.26 (0.01- 2.54e2)	0.785	0.138	0.753	0.195
			Weighted median	0.75	2.98	2.13 (0.01- 7.39e2)	0.800			
			Inverse variance weighted	0.22	2.70	0.08 (0.00-2.71e6)	0.932			
Insominia		40	MR-Egger	-0.87	1.15	0.42 (0.04-4.05)	0.456	0.506	0.167	0.503
			Weighted median	0.18	0.56	1.20 (0.40-3.63)	0.746			
			Inverse variance weighted	0.67	0.37	1.96 (0.94-4.08)	0.073			

#### 3.1.3 Sleep traits and thyroid nodules

After conducting rigorous data screening, we successfully identified a wide range of IVs for our analysis. These encompassed 87 SNPs from Daytime nap, 72 SNPs from Getting up, 73 SNPs from Sleep duration, 38 SNPs from Daytime sleepiness, 25 SNPs from Short sleep, 7 SNPs from long sleep, and 40 SNPs from Insomnia. The results revealed a potential positive correlation between Daytime nap and thyroid nodules (OR: 1.66, 95% CI: 1.07 to 2.58, P-value = 0.023, **Supplementary Table 2, Table 4**). However, subsequent MR PRESSO analysis(Distortion Test P-value = 0.023,**Table 2**) indicated the presence of horizontal pleiotropy, and after correction, the p-value became non-significant. Therefore, these findings are considered unreliable. No significant causal relationship was found between Getting up (OR: 0.84, 95% CI: 0.56 to 1.26, P-value = 0.404, **Supplementary Table 2**, **Table 4**), Sleep duration (OR: 1.31, 95% CI: 0.88 to 1.94, P-value = 0.178,**Supplementary Table 2**, **Table 4**), Daytime sleepiness (OR: 2.14, 95% CI: 0.84 to 5.48, P-value = 0.110, **Supplementary Table 2**, **Table 4**), Short sleep (OR: 1.52, 95% CI: 0.53 to 4.39, P-value = 0.724, **Supplementary Table 2**, **Table 4**), Long sleep (OR: 1.20, 95% CI: 0.07 to 21.90, P-value = 0.900, **Supplementary Table 2**, **Table 4**), and Insomnia (OR: 1.02, 95% CI: 0.48 to 2.16, P-value = 0.964, **Supplementary Table 2**, **Table 4**) in relation to thyroid nodules.

**Table 4:** Forward MR of the correlation between sleep and nodule.

(exposure)	(outcome)	Nsnp	Methods	Beta	SE	Beta/OR (95%CI)	*P* value	Heterogeneity*Q_P* value	Horizontal pleiotrop	
									Egger intercept P	*MRPresso P*
Daytime nap	nodule	87	MR-Egger	0.94	0.81	2.57 (0.52-12.63)	0.249	0.027	0.578	0.023
			Weighted median	0.45	0.31	1.57 (0.85- 2.91)	0.146			
			Inverse variance weighted	0.50	0.22	1.66 (1.07- 2.58)	0.023			
Getting up		72	MR-Egger	-0.33	0.81	0.72 (0.15-3.53)	0.682	0.005	0.838	0.523
			Weighted median	0.12	0.26	1.13 (0.68-1.88)	0.641			
			Inverse variance weighted	-0.17	0.20	0.84 (0.56-1.26)	0.404			
Sleep duration		73	MR-Egger	0.16	0.79	1.18 (0.25-5.61)	0.838	3.26e-11	0.890	0.039
			Weighted median	0.19	0.20	1.21 (0.82-1.79)	0.344			
			Inverse variance weighted	0.26	0.20	1.31 (0.88-1.94)	0.178			
Daytime sleepiness		38	MR-Egger	2.69	2.12	14.84(0.23-948.06)	0.211	0.011	0.355	0.690
			Weighted median	1.06	0.58	2.89(0.92-9.10)	0.069			
			Inverse variance weighted	0.76	0.47	2.14(0.84-5.48)	0.110			
Short sleep		25	MR-Egger	-1.08	3.02	0.34 (0.00-127.90)	0.434	0.513	0.618	0.532
			Weighted median	0.16	0.74	1.18 (0.27- 5.08)	0.822			
			Inverse variance weighted	0.42	0.53	1.52 (0.53- 4.39)	0.724			
long sleep		7	MR-Egger	-4.18	4.44	0.02 (0.00- 91.99)	0.389	0.732	0.344	0.782
			Weighted median	0.01	1.88	1.01 (0.03- 40.70)	0.993			
			Inverse variance weighted	0.18	1.48	1.20 (0.07- 21.90)	0.900			
Insominia		40	MR-Egger	0.99	1.18	2.71 (0.27-27.57)	0.403	3.26e-5	0.385	0.613
			Weighted median	0.40	0.41	1.50 (0.67- 3.37)	0.329			
			Inverse variance weighted	0.01	0.38	1.02 (0.48- 2.16)	0.964			

### 3.2 Reverse MR analysis

#### 3.2.1 Autoimmune hypothyroidism and sleep traits

When considering autoimmune hypothyroidism as the exposure in reverse MR analysis, 152 SNPs were chosen as IVs.The findings suggested a potential inverse association between Autoimmune hypothyroidism and Getting up (OR: 0.99, 95% CI: 0.98 to 1.00, P-value = 6.66e-3, **Table 2**). Additional assessments revealed heterogeneity(Q-P-value = 3.90e-10) but did not identify horizontal pleiotropy(Distortion Test P-value = 0.503, **Table 2**). As the results of MR-Egger method(OR: 1.00, 95% CI: 0.98 to 1.02, P-value = 0.742, **Table 2**) exhibited an opposing trend to that observed with the IVW method, it is therefore deemed unreliable. After adjusting for P-values, the results were not statistically significant. There were no significant associations observed between autoimmune hypothyroidism and Daytime nap (OR: 0.99, 95% CI: 1.00 to 1.01, P-value = 0.115, **Supplementary Table 2, Table 2**), Sleep duration (OR: 0.99, 95% CI: 0.98 to 1.01, P-value = 0.921, **Supplementary Table 2, Table 2**), Daytime sleepiness (OR: 1.00, 95% CI: 1.00 to 1.01, P-value = 0.103, **Supplementary Table 2, Table 2**), Short sleep (OR: 1.00, 95% CI: 1.00 to 1.01, P-value = 0.432, **Supplementary Table 2, Table 2**), Long sleep (OR: 1.00, 95% CI: 1.00 to 1.00, P-value = 0.104, **Supplementary Table 2, Table 2**), or Insomnia (OR: 1.00, 95% CI: 1.00 to 1.01, P-value = 0.468, **Supplementary Table 2, Table 2**).

#### 3.2.2 Graves’ disease and sleep traits

In the context of reverse MR analysis, utilizing graves disease as the exposure, a total of 24 SNPs were selected as IVs. However, no significant associations were found between Graves’ disease and Daytime nap (OR: 1.00, 95% CI: 0.99 to 1.01, P-value = 0.962, **Supplementary Table 2**, **Table 5**), Getting up (OR: 0.99, 95% CI: 0.98 to 1.00, P-value = 0.944, **Supplementary Table 2**, **Table 5**), Sleep duration (OR: 0.99, 95% CI: 0.99 to 1.00, P-value = 0.221, **Supplementary Table 2**, **Table 5**), Daytime sleepiness (OR: 1.00, 95% CI: 1.00 to 1.00, P-value = 0.881, **Supplementary Table 2**, **Table 5**), Short sleep (OR: 1.00, 95% CI: 1.00 to 1.00, P-value = 0.310, **Supplementary Table 2**, **Table 5**), Long sleep (OR: 1.00, 95% CI: 1.00 to 1.00, P-value = 0.863, **Supplementary Table 2**, **Table 5**), or Insomnia (OR: 1.00, 95% CI: 1.00 to 1.01, P-value = 0.118, **Supplementary Table 2**, **Table 5**).

**Table 5:** Reverse MR of the correlation between sleep and graves.

(exposure)	(outcome)	Nsnp	Methods	Beta	SE	Beta/OR (95%CI)	*P* value	Heterogeneity*Q_P* value	Horizontal pleiotrop	
									Egger intercept P	*MRPresso P*
graves	Daytime nap	24	MR-Egger	-1.17e-3	0.01	1.00 (0.98-1.02)	0.920	1..46e-7	0.927	0.220
			Weighted median	-2.45e-3	2.97e-3	1.00 (0.99-1.00)	0.409			
			Inverse variance weighted	-1.55e-4	3.32e-3	1.00 (0.99-1.01)	0.962			
	Getting up	24	MR-Egger	-9.95e-3	9.68e-3	1.00 (0.98-1.02)	0.316	0.251	0.287	0.210
			Weighted median	-1.25e-3	3.74e-3	1.00 (0.99-1.01)	0.737			
			Inverse variance weighted	1.94e-4	2.76e-3	0.99 (0.98-1.00)	0.944			
	Sleep duration	22	MR-Egger	-4.58e-3	6.40e-3	1.00 (0.98-1.01)	0.483	0.059	0.885	0.075
			Weighted median	-6.38e-3	3.79e-3	0.99 (0.99-1.00)	0.091			
			Inverse variance weighted	-3.76e-3	3.08e-3	1.00 (0.99-1.00)	0.221			
	Daytime sleepiness	22	MR-Egger	9.47e-4	2.63e-3	1.00 (1.00-1.01)	0.722	0.198	0.744	0.179
			Weighted median	-1.39e-3	1.73e-3	1.00 (1.00-1.00)	0.421			
			Inverse variance weighted	1.89e-4	1.26e-3	1.00 (1.00-1.00)	0.881			
	Short sleep	22	MR-Egger	7.86e-4	2.92e-3	1.00 (0.99-1.01)	0.791	0.803	0.015	0.017
			Weighted median	7.56e-4	1.70e-3	1.00 (1.00-1.00)	0.656			
			Inverse variance weighted	1.42e-3	1.40e-3	1.00 (1.00-1.00)	0.310			
	long sleep	22	MR-Egger	1.90e-3	1.62e-3	1.00 (1.00-1.01)	0.254	0.521	0.164	0.544
			Weighted median	2.18e-4	1.20e-3	1.00 (1.00-1.00)	0.856			
			Inverse variance weighted	-1.38e-4	8.01e-4	1.00 (1.00-1.00)	0.863			
	Insominia	22	MR-Egger	9.50e-4	3.28e-3	0.99 (0.98-1.01)	0.775	0.358	0.599	0.395
			Weighted median	9.66e-4	2.21e-3	1.00 (0.99-1.01)	0.662			
			Inverse variance weighted	2.47e-3	1.58e-3	1.00 (1.00-1.01)	0.118			

#### 3.2.3 Thyroid nodules and sleep traits

**Figure 2:**
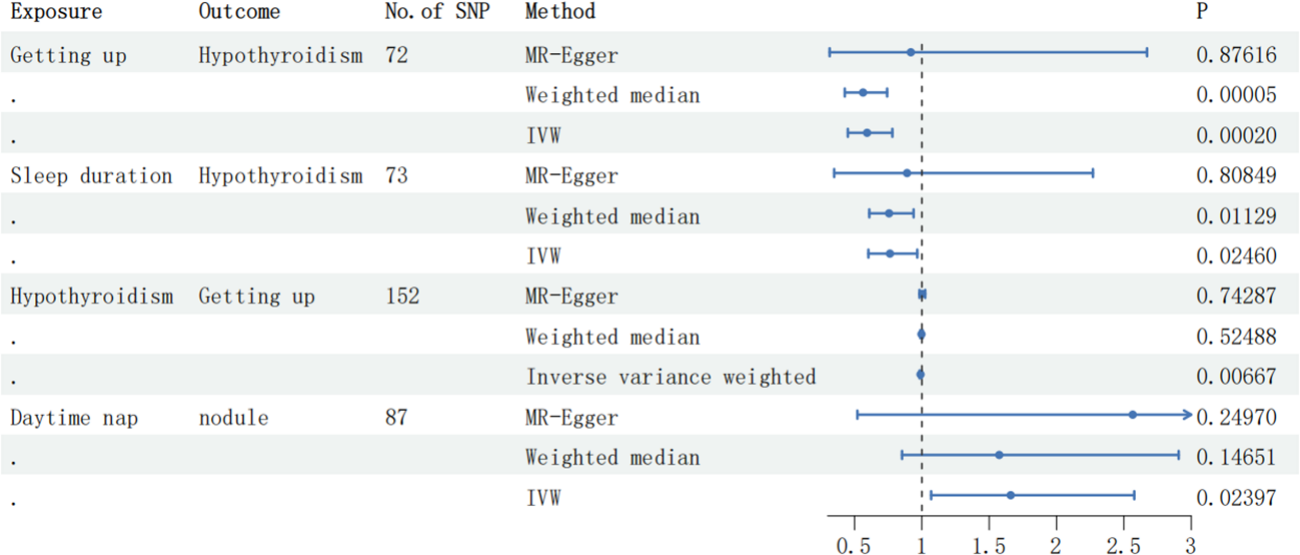
The forest plot displaying the correlation results in the MR analysis. SNP, single-nucleotide polymorphism; IVW, inverse variance weighted.

By employing reverse MR analysis, incorporating Thyroid nodules as the focal exposure factor, a total of 48 SNP markers were selected as IVs. Nevertheless, no statistically significant associations were observed between Thyroid nodules and various sleep-related outcomes, including Daytime nap (OR: 1.00, 95% CI: 0.99 to 1.00, P-value = 0.078, **Supplementary Table 2**, **Table 6**), Getting up (OR: 0.99, 95% CI: 0.98 to 1.00, P-value = 0.456, **Supplementary Table 2**, **Table 6**), Sleep duration (OR: 1.00, 95% CI: 0.99 to 1.00, P-value = 0.120, **Supplementary Table 2, Table 6****Table 6**), Daytime sleepiness (OR: 1.00, 95% CI: 1.00 to 1.00, P-value = 0.695, **Supplementary Table 2**, **Table 6**), Short sleep (OR: 1.00, 95% CI: 1.00 to 1.00, P-value = 0.452, **Supplementary Table 2**, **Table 6**), Long sleep (OR: 1.00, 95% CI: 1.00 to 1.00, P-value = 0.644, **Supplementary Table 2**, **Table 6**), or Insomnia (OR: 1.00, 95% CI: 1.00 to 1.01, P-value = 0.375, **Supplementary Table 2**, **Table 6**). **Figure 2** shows the forest plot of positive results related to MR analysis.

**Table 6:** Reverse MR of the correlation between sleep and nodule.

(exposure)	(outcome)	Nsnp	Methods	Beta	SE	Beta/OR (95%CI)	*P* value	Heterogeneity*Q_P* value	Horizontal pleiotrop	
									Egger intercept P	*MRPresso P*
nodule	Daytime nap	48	MR-Egger	3.99e-3	5.4e-3	1.00 (0.99-1.01)	0.463	0.168	0.132	0.200
			Weighted median	-2.93e-3	2.80e-3	1.00 (0.99-1.00)	0.295			
			Inverse variance weighted	-3.66e-3	2.08e-3	1.00 (0.99-1.00)	0.078			
	Getting up	48	MR-Egger	-5.23e-3	8.19e-3	1.00 (0.98-1.02)	0.526	0.017	0.699	0.330
			Weighted median	-2.24e-3	3.74e-3	1.00 (0.99-1.01)	0.548			
			Inverse variance weighted	-2.29e-4	3.07e-3	0.99 (0.98-1.00)	0.456			
	Sleep duration	45	MR-Egger	-1.91e-4	6.48e-3	1.00 (0.98-1.01)	0.976	0.139	0.485	0.144
			Weighted median	-4.03e-3	3.94e-3	0.99 (0.99-1.00)	0.306			
			Inverse variance weighted	-4.31e-3	2.77e-3	1.00 (0.99-1.00)	0.120			
	Daytime sleepiness	45	MR-Egger	1.65e-3	3.03e-3	1.00 (1.00-1.01)	0.587	0.092	0.676	0.104
			Weighted median	5.53e-4	1.77e-3	1.00 (1.00-1.00)	0.754			
			Inverse variance weighted	5.06e-4	1.29e-3	1.00 (1.00-1.00)	0.695			
	Short sleep	45	MR-Egger	-5.28e-4	3.25e-3	1.00 (0.99-1.01)	0.871	0.002	0.594	0.355
			Weighted median	-4.27e-4	1.85e-3	1.00 (1.00-1.00)	0.818			
			Inverse variance weighted	1.04e-3	1.38e-3	1.00 (1.00-1.00)	0.452			
	long sleep	45	MR-Egger	2.03e-3	1.95e-3	1.00 (1.00-1.01)	0.301	0.272	0.174	0.289
			Weighted median	-2.02e-4	1.21e-3	1.00 (1.00-1.00)	0.867			
			Inverse variance weighted	-3.91e-4	8.48e-4	1.00 (1.00-1.00)	0.644			
	Insominia	45	MR-Egger	-5.99e-3	4.96e-3	0.99 (0.98-1.00)	0.234	5.10e-5	0.083	<0.001
			Weighted median	2.24e-4	2.52e-3	1.00 (1.00-1.01)	0.374			
			Inverse variance weighted	1.94e-3	2.18e-3	1.00 (1.00-1.01)	0.375			

### 3.3 F-statistics and Visualization of MR

**Figure 3:**
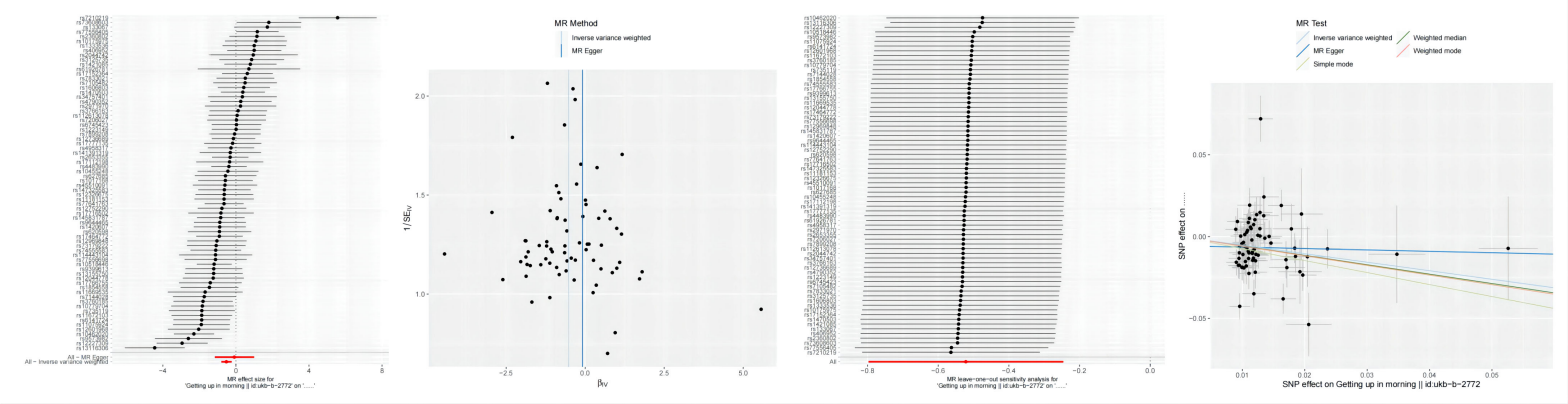
From left to right includes forest plots, funnel plots,leave-one-out plots and scatter plots, showcasing the MR Effect., the MR Effect of Getting up on Autoimmune hypothyroidism;.

The calculation of F-statistics for each valid IV revealed that none of them fell below 10, affirming their appropriateness for analysis (**Supplementary Table 1**). **Figure 3** incorporates a range of graphical representations, namely forest plots, funnel plots, leave-one-out plots, and scatter plots, effectively illustrating the MR Effect. The scatter plot depicted in **Figure 3** which displays a negative correlation trend between Autoimmune hypothyroidism and Getting up. Furthermore, the symmetrical funnel plots indicate result stability, while the forest plots enable examination of the effects associated with each SNP. Lastly, the leave-one-out analysis validates the significance of the obtained results.

## 4 Discussion

Within the scope of this dual-directional MR analysis research, we explored the connections between seven distinct sleep characteristics (Daytime nap, Getting up, Sleep duration, Daytime sleepiness, Short sleep, Long sleep, and Insomnia) and Graves’ disease, Autoimmune hypothyroidism and Thyroid nodules. The outcomes of our investigation demonstrated a significant inverse relationship between Getting up and hypothyroidism, indicating that Getting up may function as a protective factor against hypothyroidism. However, the potential correlation between sleep duration and hypothyroidism did not reach statistical significance following P-value adjustment. Furthermore, no interrelationships were identified between Graves disease, thyroid nodules, and sleep characteristics.

**Figure 4:**
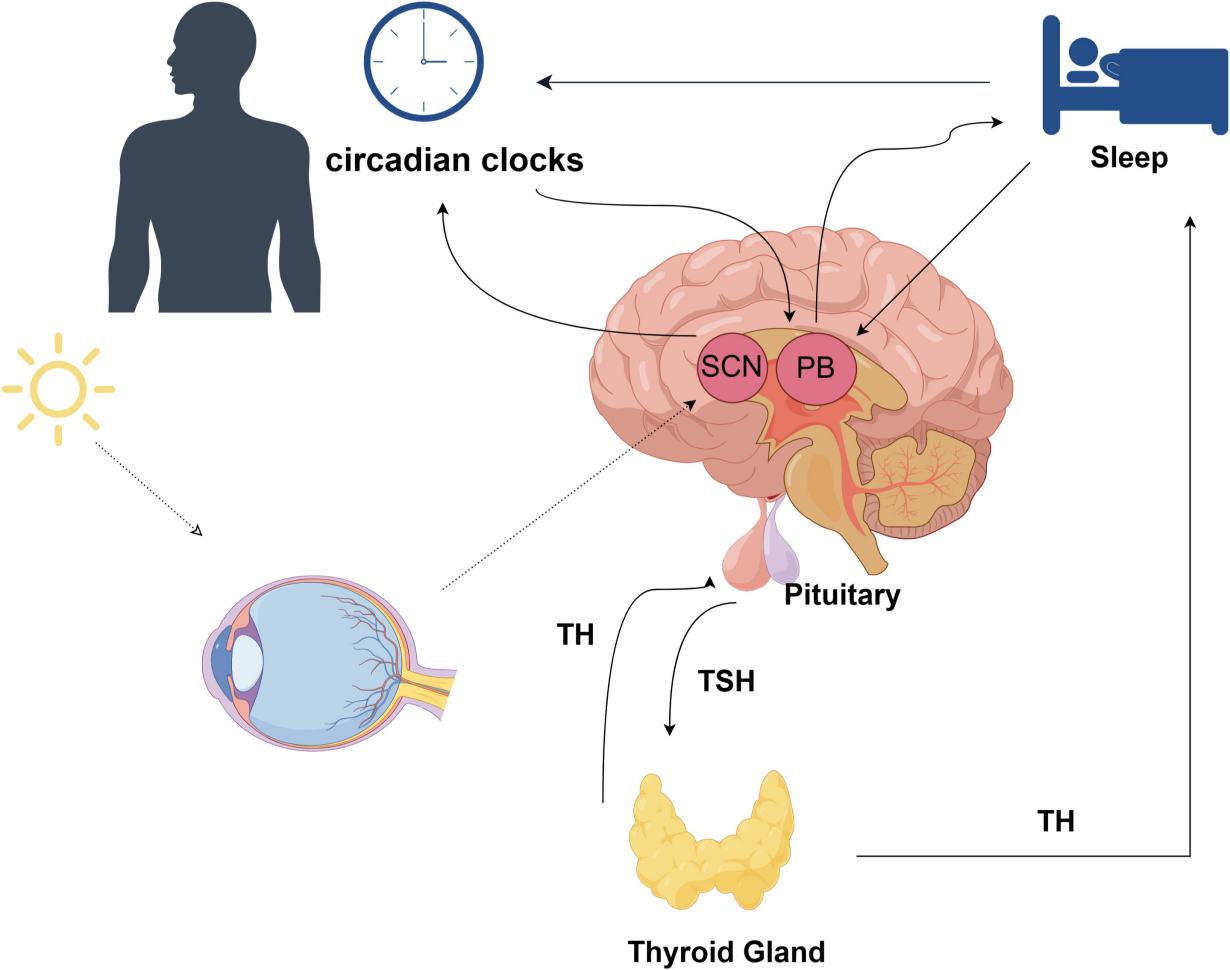
The association between sleep and the hypothalamic-pituitary-thyroid (HPT) axis; TH, thyroid hormone; TSH, thyroid stimulating hormone; SCN, Suprachiasmatic Nucleus; PB, Pineal Body;.

There exists a dual association between sleep and the hypothalamic-pituitary-thyroid (HPT) axis (**Figure 4**), wherein these interconnected homeostatic mechanisms mutually depend on each other to ensure efficient physiological performance. Notably, the characteristics and duration of sleep play a crucial role in modulating the circadian pattern of TSH and thyroid hormone secretion (45). The anatomical foundation for the diurnal variations in TRH synthesis and secretion is formed by the neural connections originating from the suprachiasmatic nucleus (SCN) and terminating at thyrotropin-releasing hormone (TRH) neurons in the paraventricular nucleus (46–48). Consequently, these neural projections play a pivotal role in regulating rhythmic TSH secretion from the pars distalis.There is additional evidence indicating that sleep exerts an influence on thyroid hormone secretion, resulting in a reduction of the circadian rhythm amplitude for TSH and subsequent levels of thyroid hormones (49). Another study indicated that post-sleep restriction recovery sleep exhibited a more substantial TSH suppression compared to regular sleep, thereby illustrating the unique impact of sleep on TSH secretion (50). The relationship between TH and the sleep-wake cycle remains unclear; however, there is evidence of the influence of circadian TH levels on sleep, where elevated TH levels are associated with shortened sleep duration. It is suggested that the elevation of T4 resulting from sleep deprivation serves as an adaptive physiological response to promote wakefulness (51).

Previous observational studies have suggested a potential association between hypothyroidism and sleep, indicating a possible correlation. A population-based study conducted in China proposed that subclinical hypothyroidism serves as a risk factor for poor sleep quality (21). Furthermore, results from another retrospective study conducted at a single center revealed a higher occurrence of early chronotypes among women diagnosed with hypothyroidism within the study’s cohort (52). Limited evidence exists regarding the association between sleep and hypothyroidism, and our study presents, for the first time, a novel causal relationship between Getting up and Autoimmune hypothyroidism. We propose that Getting up may serve as a protective factor against Autoimmune hypothyroidism, providing new evidence from a genetic variation perspective. Sleep disturbance is a characteristic feature and frequently reported symptom among individuals diagnosed with hyperthyroidism, attributable to elevated thyroid hormone production or thyrotoxicosis resulting from the release of excessive TH levels during a destructive or inflammatory process affecting the thyroid gland (53, 54). Earlier investigations into individuals with clinical hyperthyroidism revealed an increase in sleep latency, accompanied by a reduction in total sleep time and SWS (54). Additionally, a separate study by Kronfol et al. confirmed a decrease in sleep efficiency, duration, and SWS (55). The results of a retrospective observational study yielded opposing results, suggesting that there were no discernible variations in subjective measures of sleep between individuals diagnosed with overt hyperthyroidism and euthyroid controls (56). Our study findings did not establish any discernible connection between Graves’ disease itself and sleep, leading us to conclude that there is no direct causal relationship between the two. Instead, it is plausible that the observed manifestations of increased sympathetic nervous system activity, such as heightened neural excitability resulting from thyrotoxicosis, may mediate their association. Analysis of data from a community-based population study indicated that individuals with shorter periods of sleep at night were more likely to exhibit thyroid nodules (57). These findings have prompted speculation about potential disruptions in the circadian gene expression patterns within the thyroid gland (58). Notably, alterations in circadian rhythm genes, particularly elevated expressions of CLOCK and BMAL1, along with decreased expression of CRY2, were predominantly observed in malignant nodules (59). The results of our study did not establish a causal link between thyroid nodules and sleep traits, highlighting the importance of conducting further exploratory investigations to gain deeper insights into this relationship.

Our study boasted several notable strengths that contribute to its validity. Firstly, it was the first of its kind to investigate the causal association between sleep traits and AITD as well as thyroid nodules using bidirectional two-sample Mendelian randomization. Notably, the use of distinct databases for the exposure and outcome datasets helped mitigate concerns regarding potential sample overlap. Moreover, we employed IVs consisting of SNPs with strong associations (P<5e-8) and high intensity (F-statistics > 10), ensuring greater comparability between the exposure and outcome groups. This approach lends greater credibility to our findings. Furthermore, we conducted an extensive sensitivity analysis to assess the robustness of our conclusions. However, it is important to acknowledge the limitations of our research. Firstly, As not all genetic variants and traits adhere to Mendel’s second law, wherein genes act independently and randomly, the utilization of genes as IVs in studies may be susceptible to bias arising from weak instruments, population stratification, and developmental compensation. Further validation using larger GWAS data is warranted. Furthermore, our study did not stratify the AITD patient population by gender, which may introduce heterogeneity due to variations in health status, age, or gender. Additionally, it is important to acknowledge that our study primarily included individuals of European descent, which limits the generalizability of our findings to a global population. Thirdly, the data on sleep characteristics were obtained through questionnaire surveys, making it difficult to avoid subjective biases and inevitable misclassification. Moreover, despite implementing measures to identify and eliminate outlier variants, we cannot exclude the possibility that horizontal pleiotropy influenced our results.

## 5 Conclusion

To summarize, the results of our study reveal a notable cause-and-effect relationship between Getting up and Autoimmune hypothyroidism, indicating its potential role as a protective factor against this condition. However, no causal connection was observed between sleep traits and Graves’ disease or Thyroid nodules. The genetic variation approach employed in our research offers fresh evidence that complements the conclusions drawn from previous observational studies. Further verification through larger-scale GWAS investigations is necessary.

## Data availability statement

The original contributions presented in the study are included in the article/**Supplementary Table Material**. Further inquiries can be directed to the corresponding author.

## Author contributions

SW: Conceptualization, Data curation, Formal analysis, Methodology, Project administration, Software, Visualization, Writing – original draft, Writing – review & editing. KW: Data curation, Formal analysis, Methodology, Project administration, Software, Supervision, Writing – review & editing. SL: Funding acquisition, Project administration, Resources, Supervision, Validation, Writing – review & editing. XC: Conceptualization, Methodology, Software, Supervision, Validation, Writing – review & editing.
